# Supplementary figures and images for: TDP2 drives immune evasion and metastatic progression in prostate cancer
Source: PLoS One. 2026 Jan 2;21(1):e0339607. doi: 10.1371/journal.pone.0339607 (PMC12758750; doi:10.1371/journal.pone.0339607)

**A**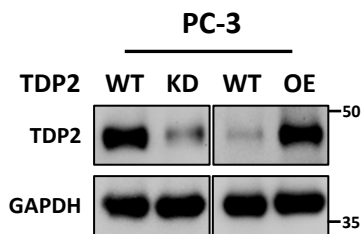**B**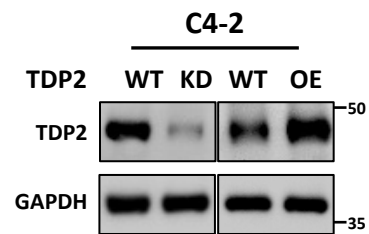**C**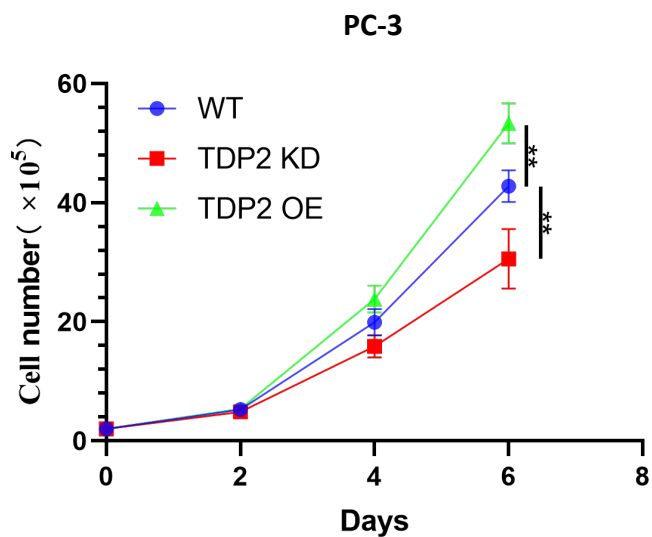**D**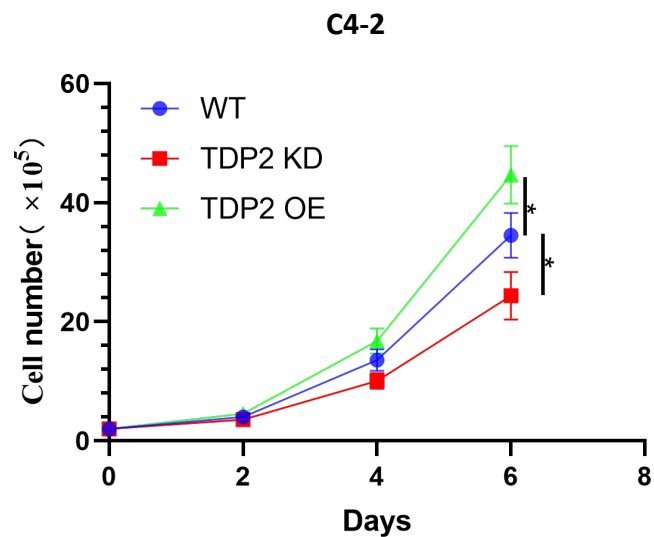**E**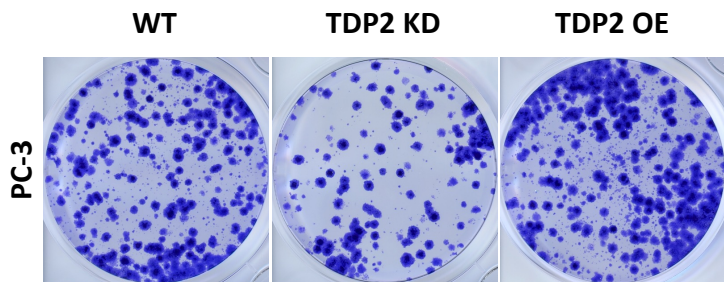**F**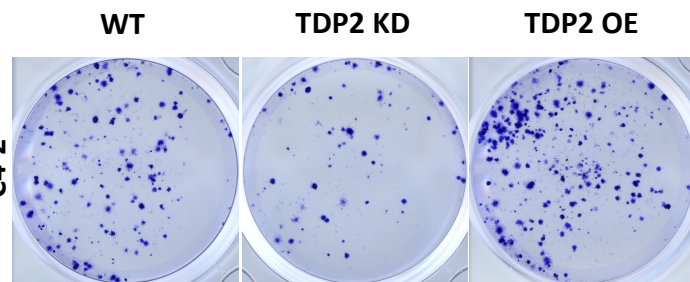**G**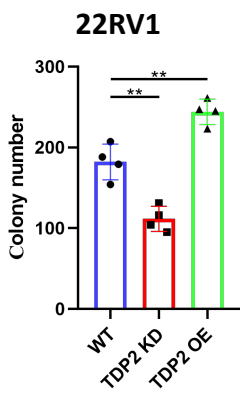**H**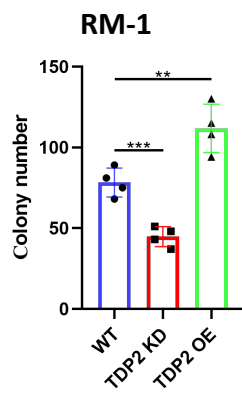**I**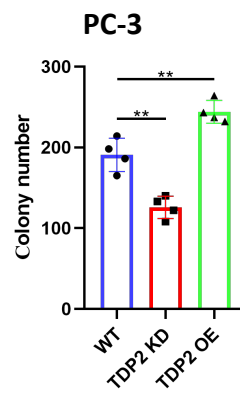**J**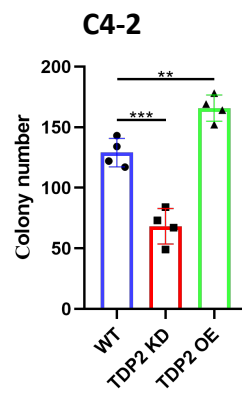

Supplement: S1 Fig — Western blot data demonstrating changes in TDP2 protein concentration in PC-3 (A) and C4-2 (B) cells transfected with shRNA-TDP2 or TDP2. Cell growth curves (C, D) and colony formation assays (E, F) were performed to assess the proliferation of TDP2 KD or TDP2 OE transduced PC-3 and C4-2 cells. Quantification of the colony number in 22RV1 (G), RM-1 (H), PC-3 (I) and C4-2 (J) cells. Data were presented as means ± standard deviations. P values were determined by the one-way ANOVA, *P < 0.05, **P < 0.01, ***P < 0.001. (PDF) [file pone.0339607.s001.pdf]

**A****22RV1****WT****TDP2 KD****TDP2 OE****0H**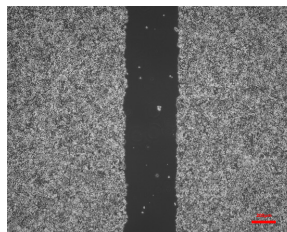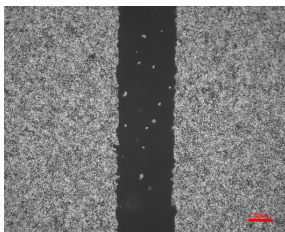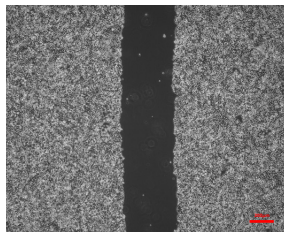**72H**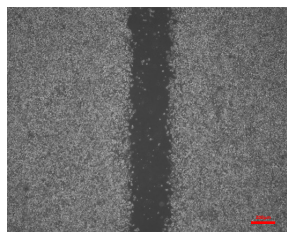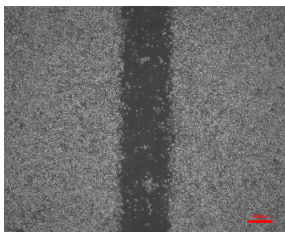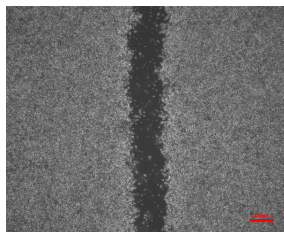**Relative migration(%)**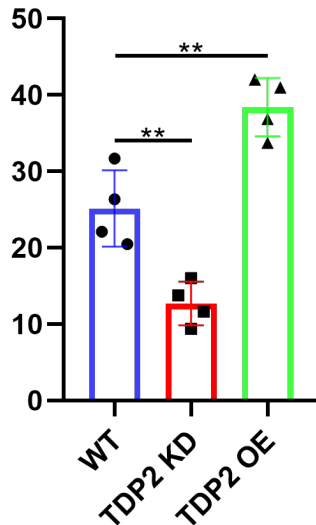**B****RM-1****WT****TDP2 KD****TDP2 OE****0H**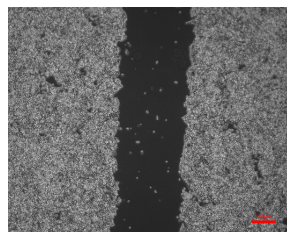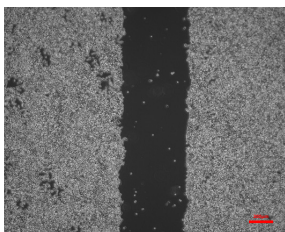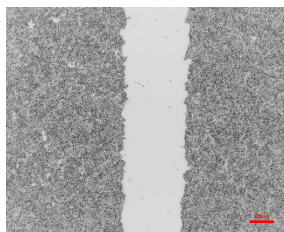**72H**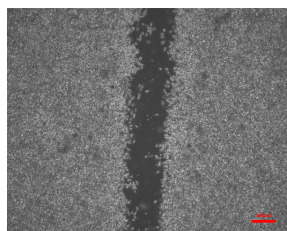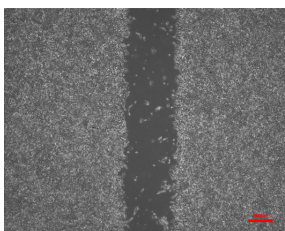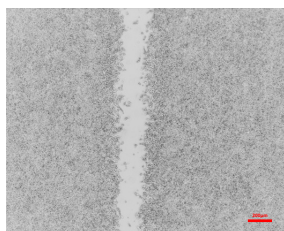**Relative migration(%)**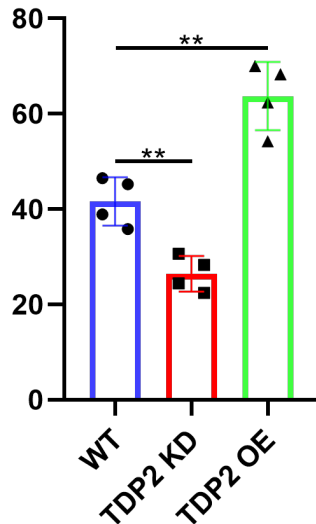

Supplement: S2 Fig — Cell wound healing assays in 22RV1 (A) and RM-1 (B) cells with overexpression and knockout of TDP2. The width of cell wound healing was measured (n = 4/group). WT, wild type; TDP2 OE, TDP2 overexpression; TDP2 KD, TDP2 knockdown. Data were presented as means ± standard deviations. P values were determined by the one-way ANOVA, **P < 0.01. (PDF) [file pone.0339607.s002.pdf]

**A**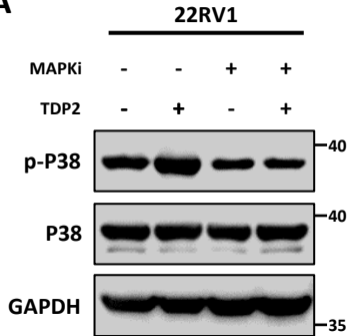**B**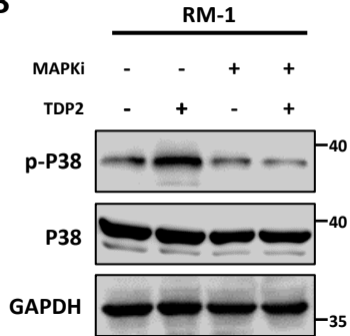

Supplement: S3 Fig — 22RV1(A) and RM-1 (B) WT/TDP2 OE cells were treated by 30 μM Adezmapimod. The expression of p38 and the corresponding phosphorylated forms was detected by Western blot. (PDF) [file pone.0339607.s003.pdf]

**A****WT**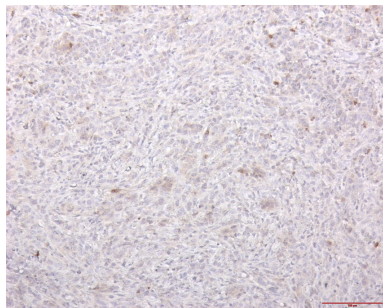**TDP2 KD**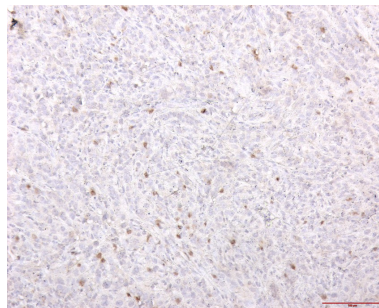**B**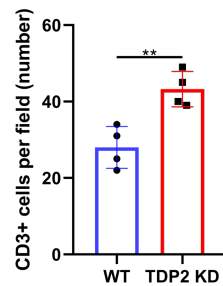**C****WT**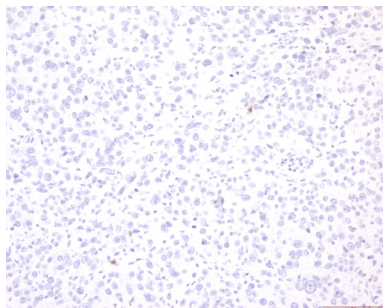**TDP2 KD**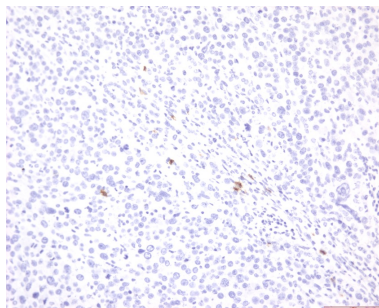**D**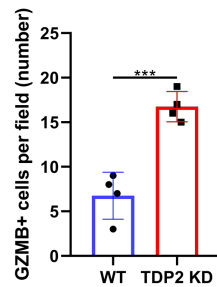

Supplement: S4 Fig — Pathology studies show CD3 (A) and GZMB (C) expression in mice across different groups. CD3 (B) and GZMB (D) positivity analysis in the RM-1 model (n = 4). The bars represent 100 μm. Data are presented as means ± standard deviations. Error bars indicate SD obtained from four independent experiments. P values were determined using the unpaired Student’s t-test, ns = not significant, **P < 0.01, ***P < 0.001. (PDF) [file pone.0339607.s004.pdf]

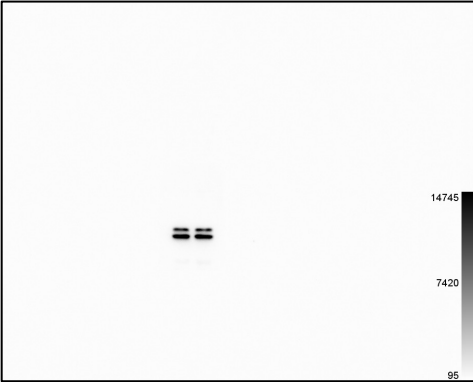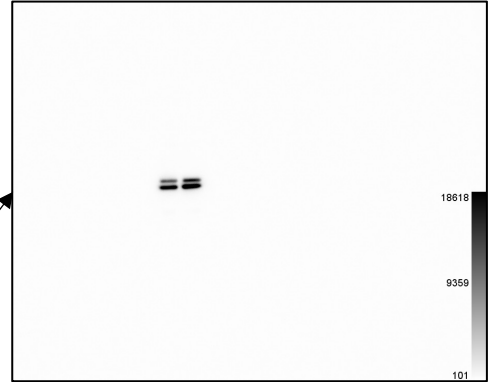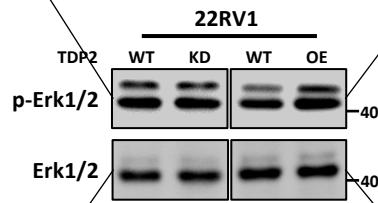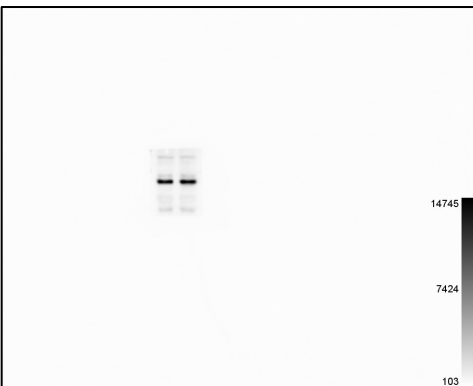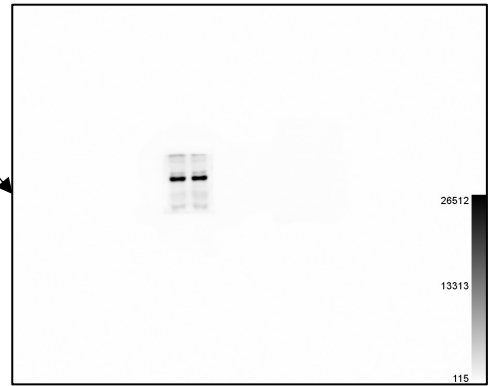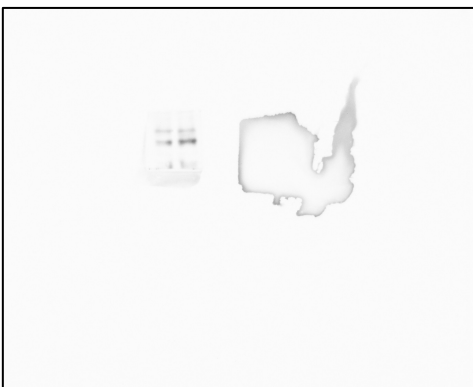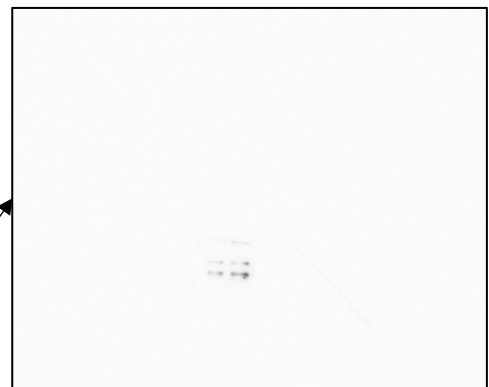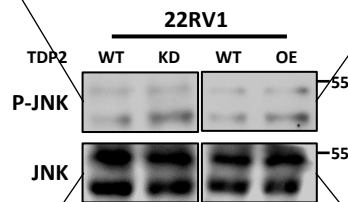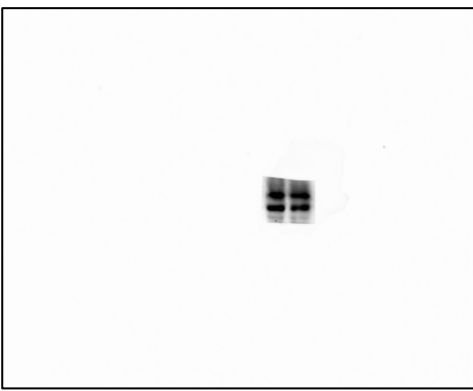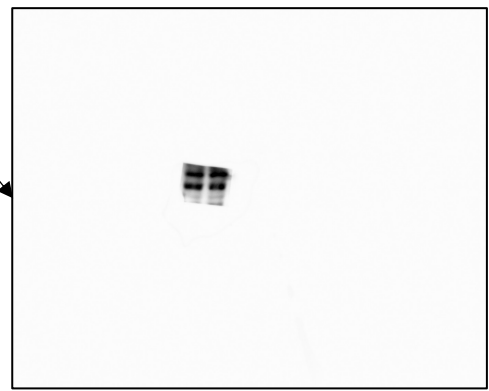

Supplement: S5 Fig — (PDF) [file pone.0339607.s005.pdf]

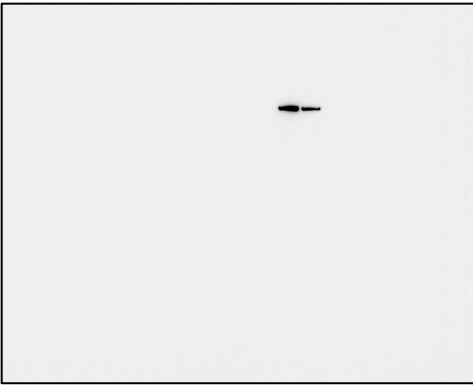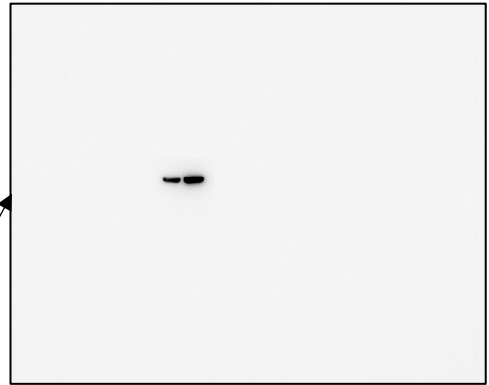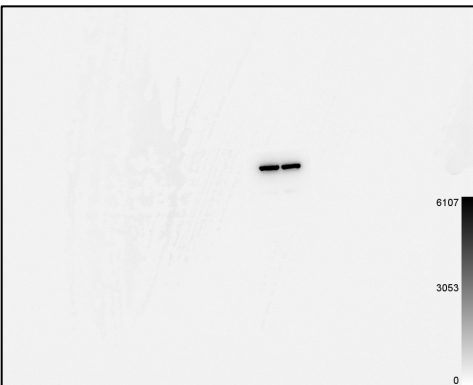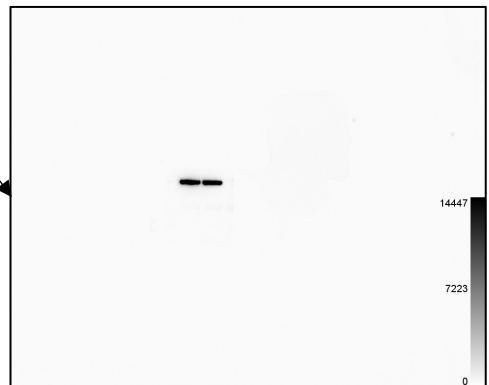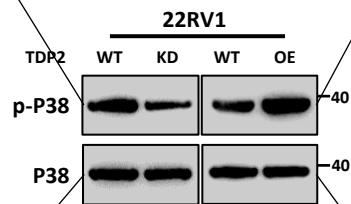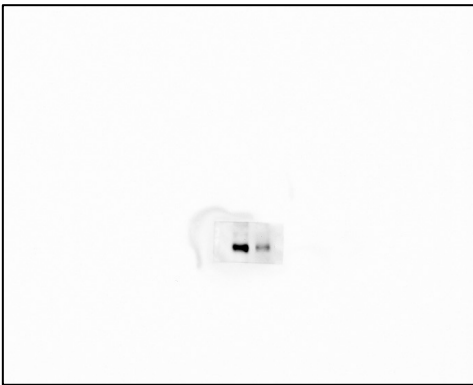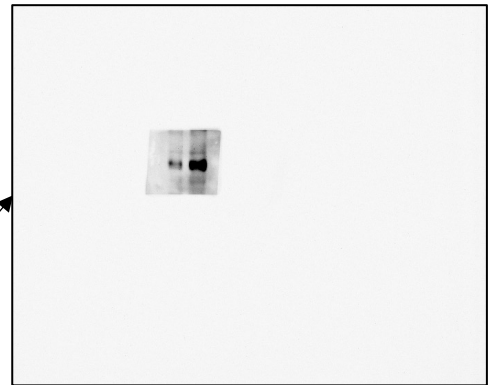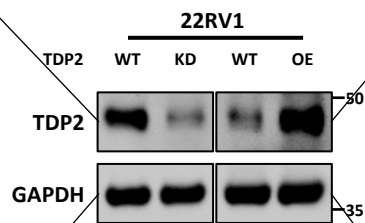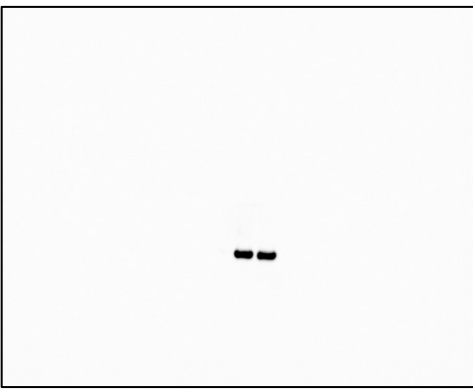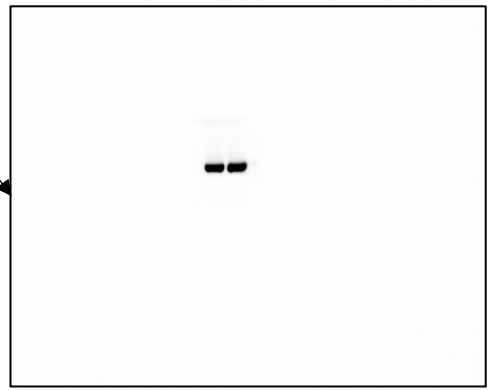

Supplement: S6 Fig — (PDF) [file pone.0339607.s006.pdf]

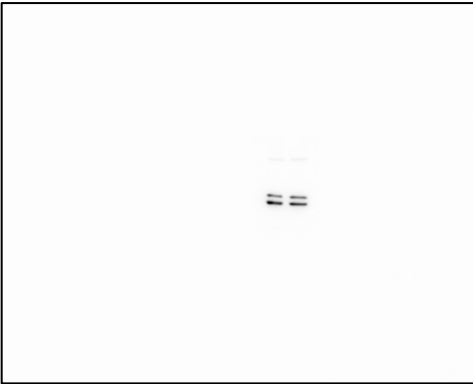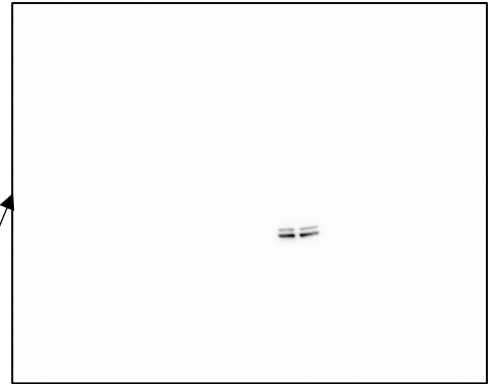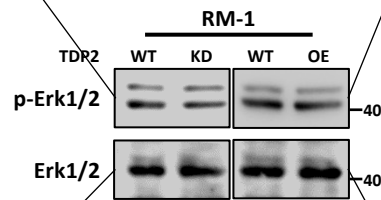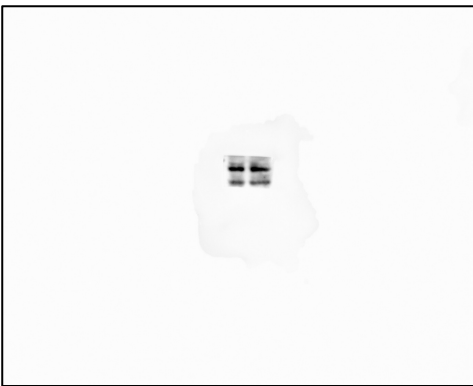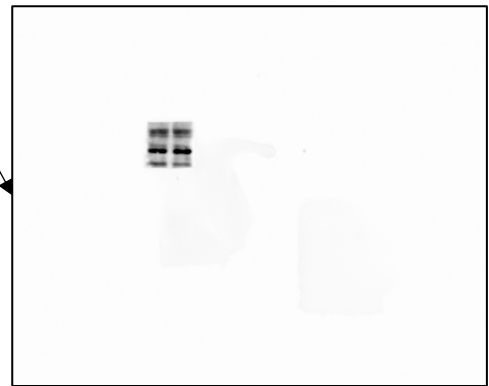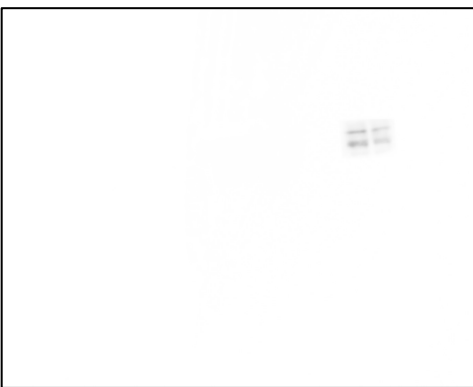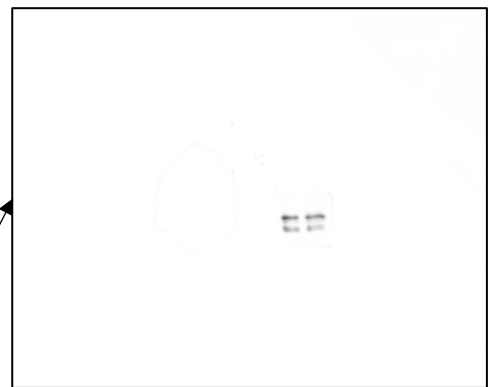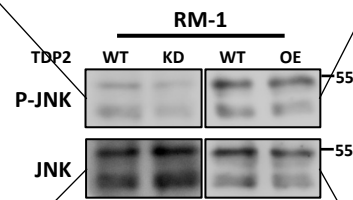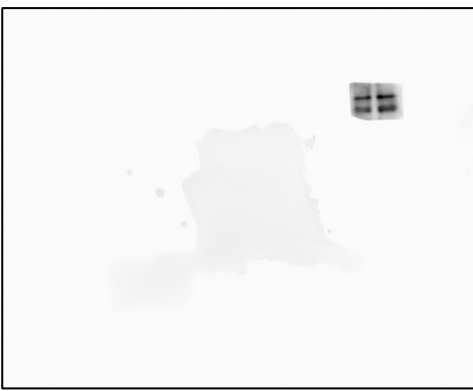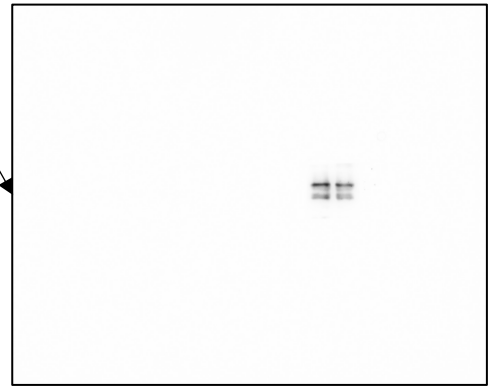

Supplement: S7 Fig — (PDF) [file pone.0339607.s007.pdf]

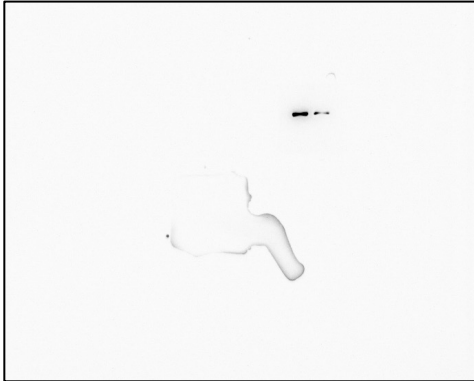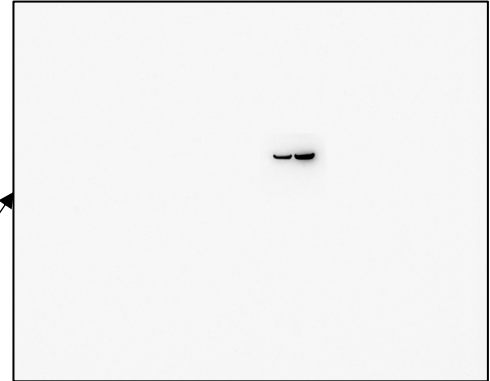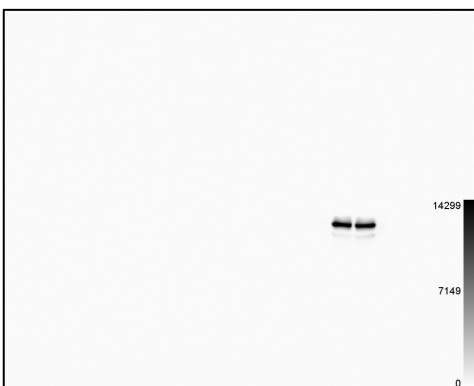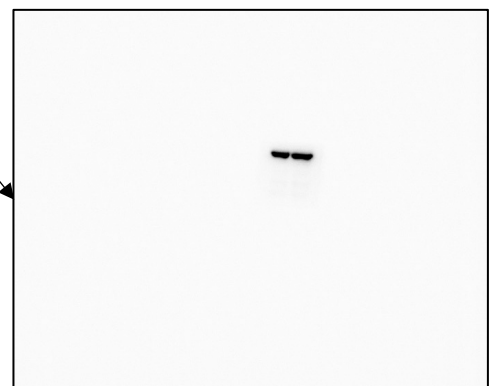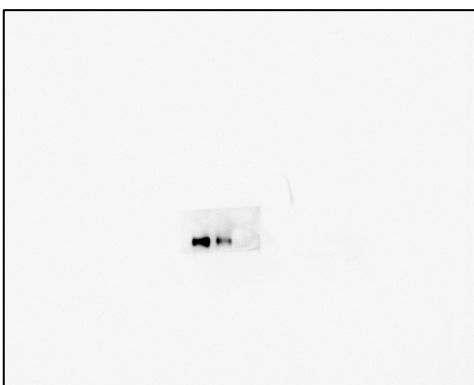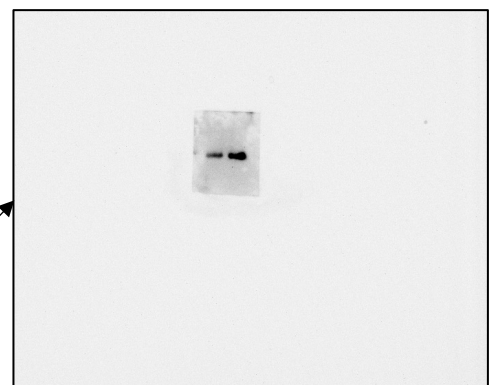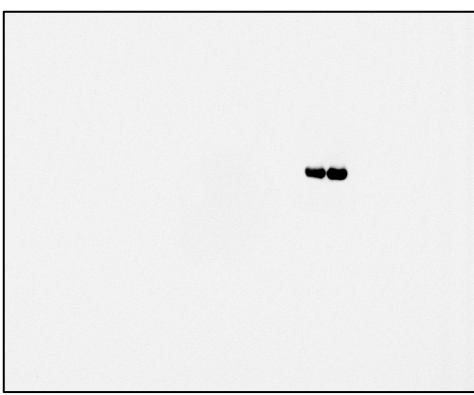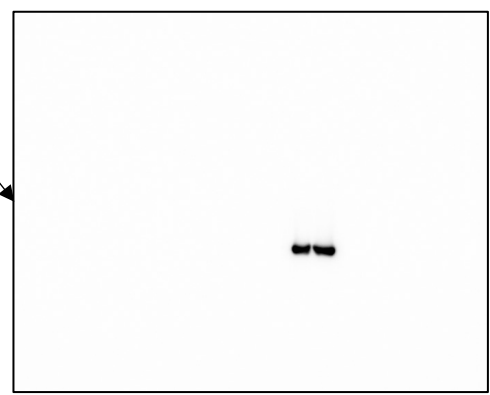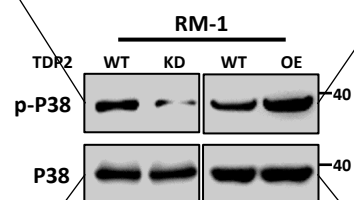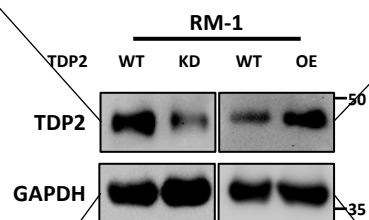

Supplement: S8 Fig — (PDF) [file pone.0339607.s008.pdf]

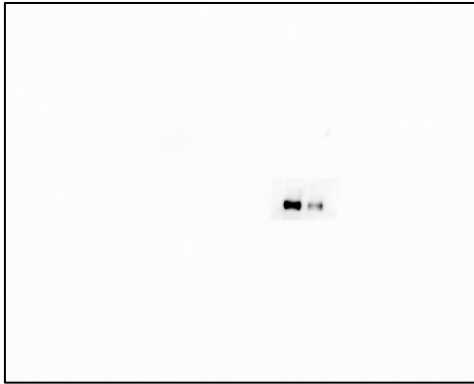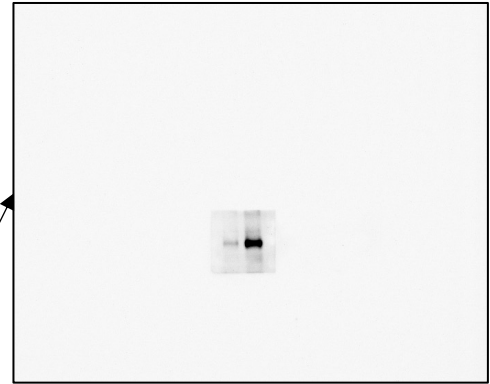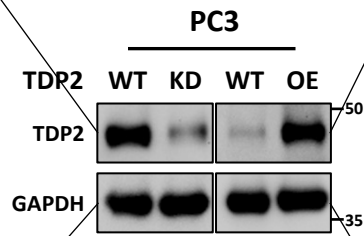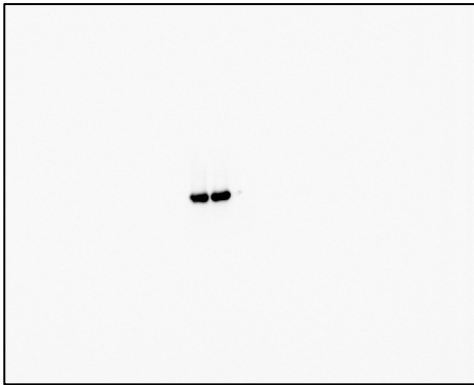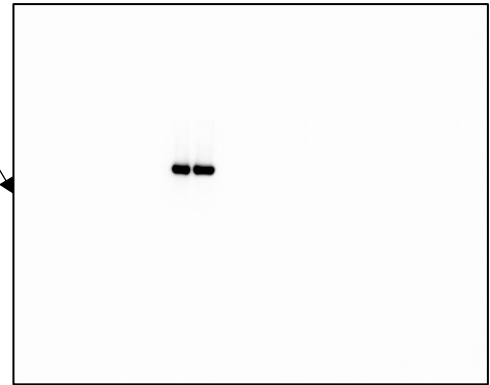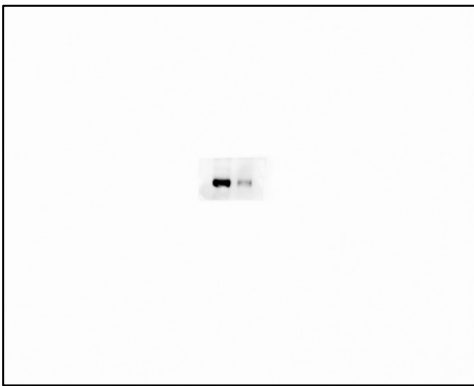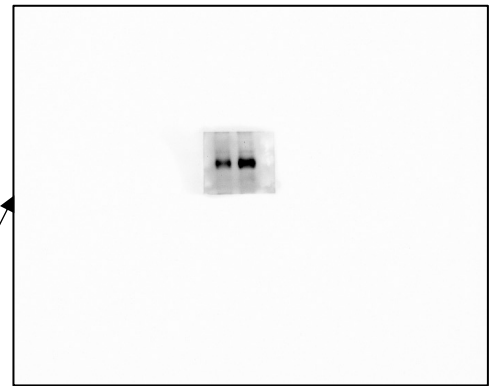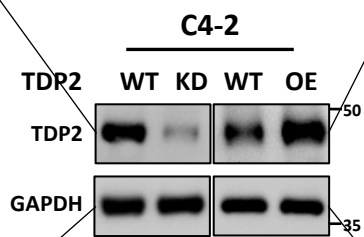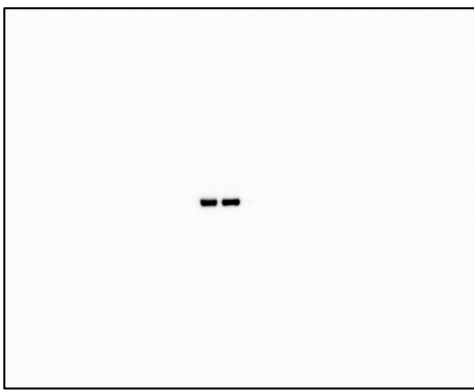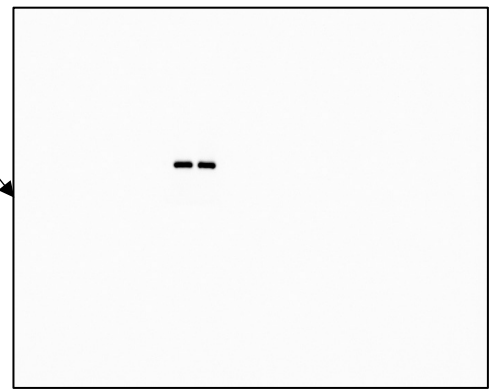

Supplement: S9 Fig — (PDF) [file pone.0339607.s009.pdf]

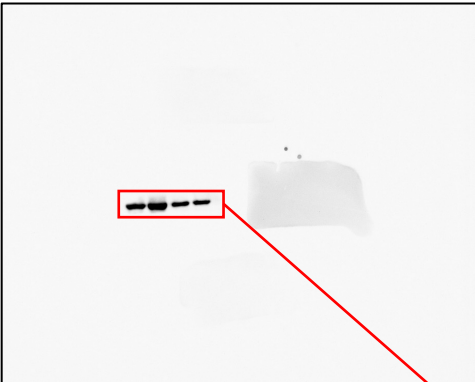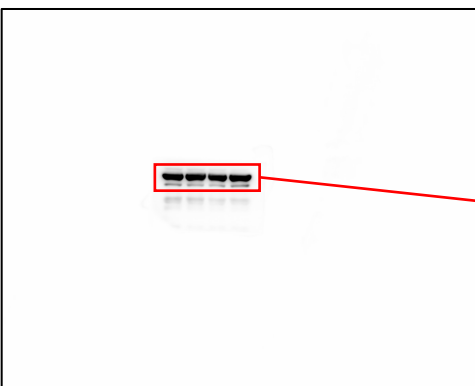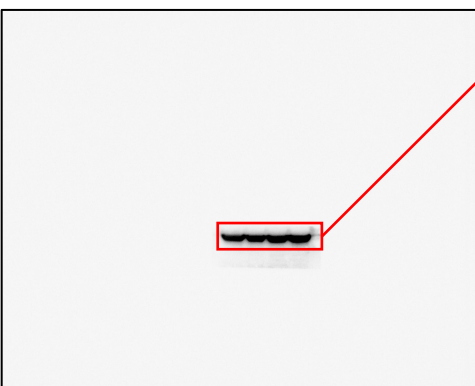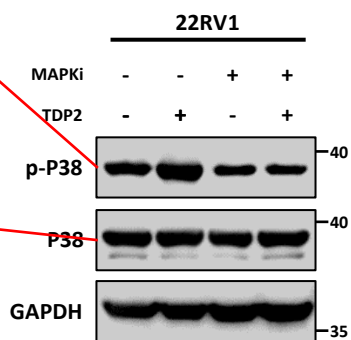

Supplement: S10 Fig — (PDF) [file pone.0339607.s010.pdf]

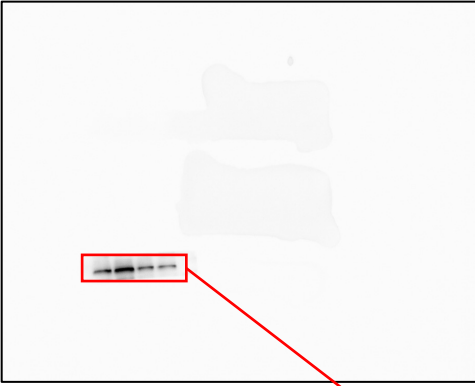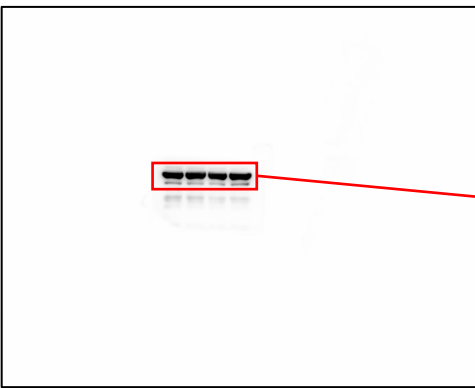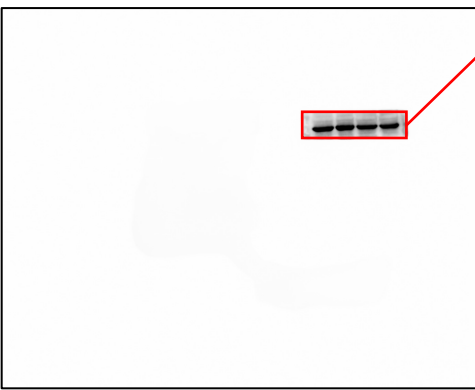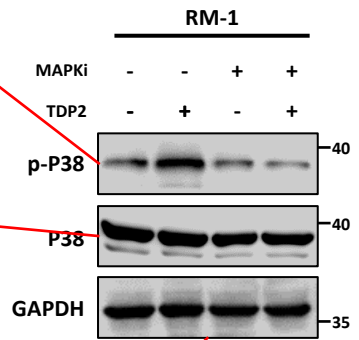

Supplement: S11 Fig — (PDF) [file pone.0339607.s011.pdf]
