# Supplementary material for: TDP2 drives immune evasion and metastatic progression in prostate cancer
Source: PLoS One. 2026 Jan 2;21(1):e0339607. doi: 10.1371/journal.pone.0339607 (PMC12758750; doi:10.1371/journal.pone.0339607)

Figure 4A

p-Erk1/2 (42,44KD)

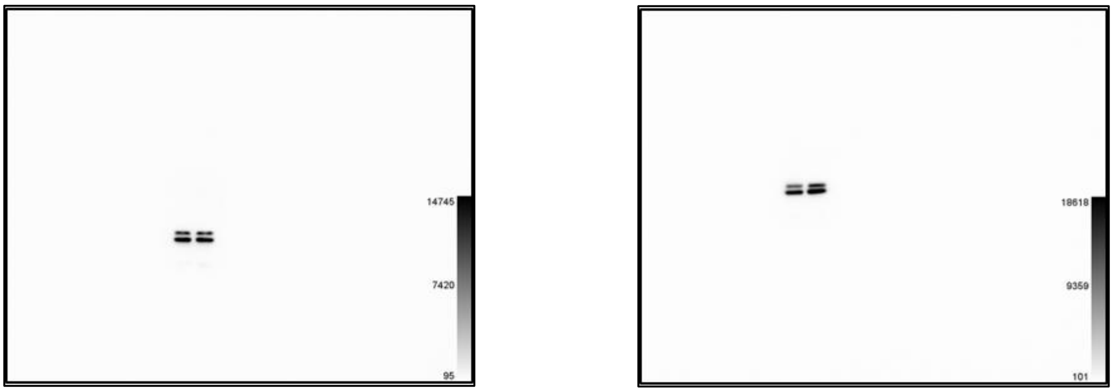

Erk1/2 (42,44KD)

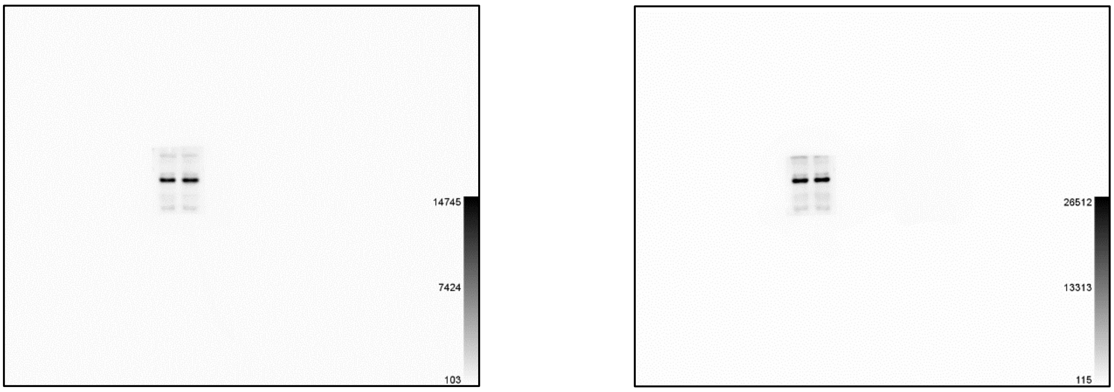

P-JNK (46,54KD)

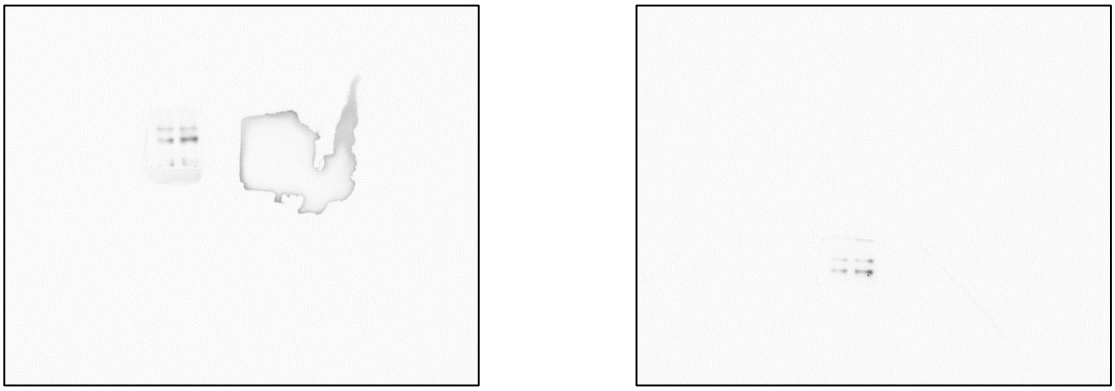

JNK (46,54KD)

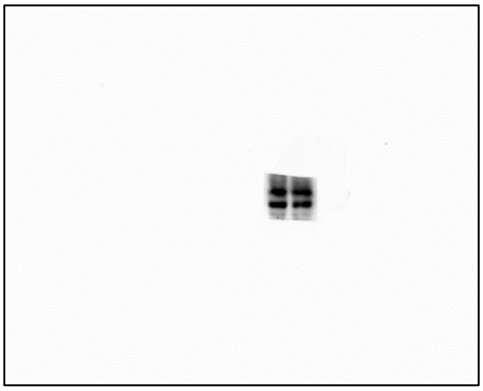

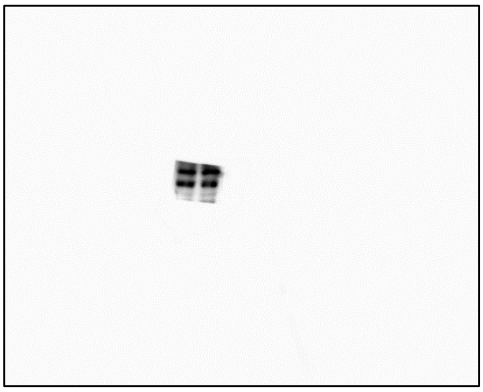

p-P38 (40KD)

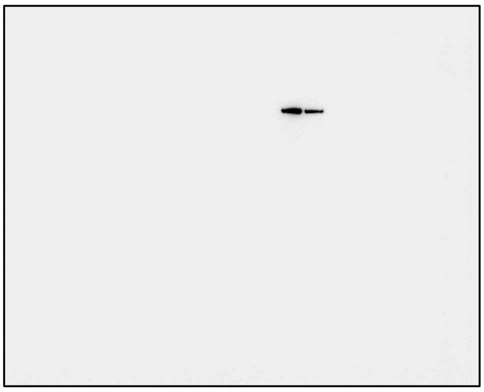

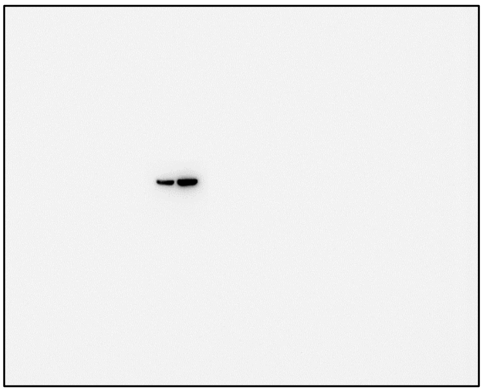

P38 (40KD)

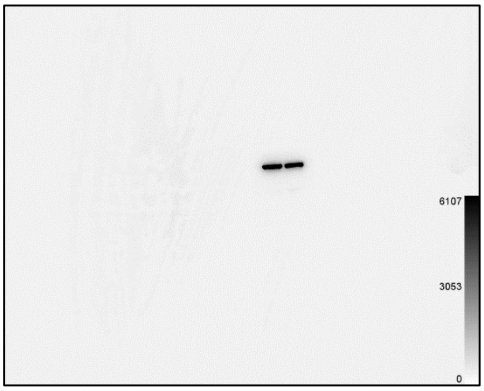

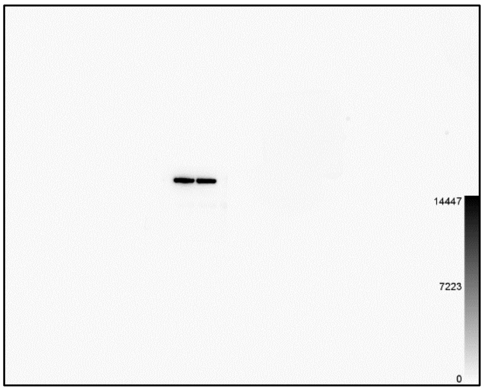

TDP2 (41KD)

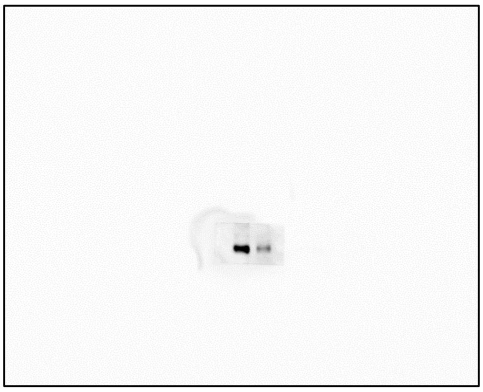

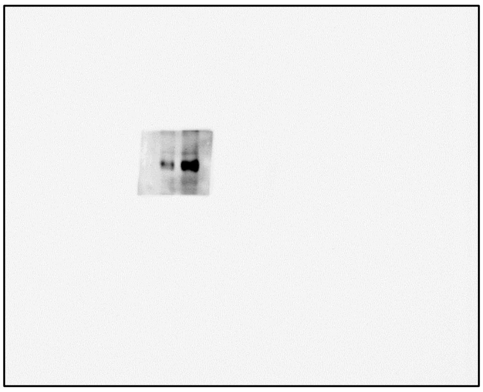

GAPDH (37KD)

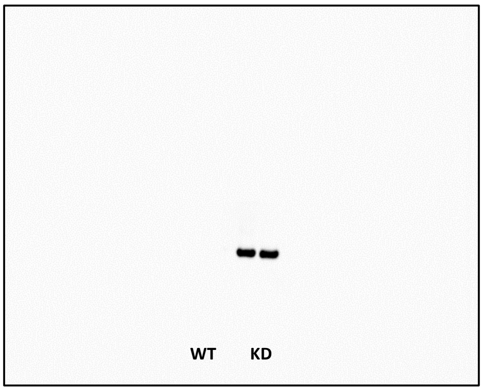

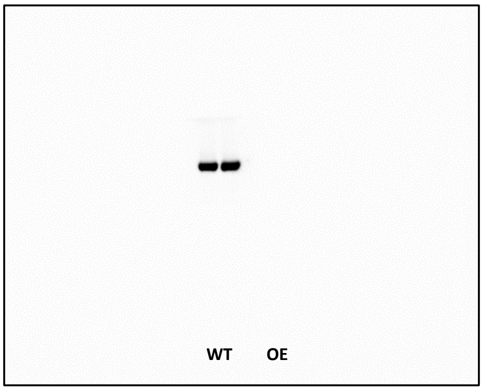

Figure 4C

p-Erk1/2 (42,44KD)

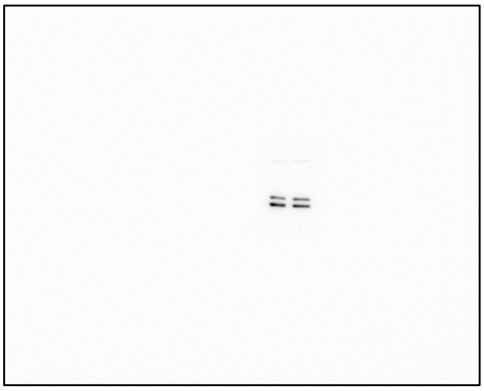

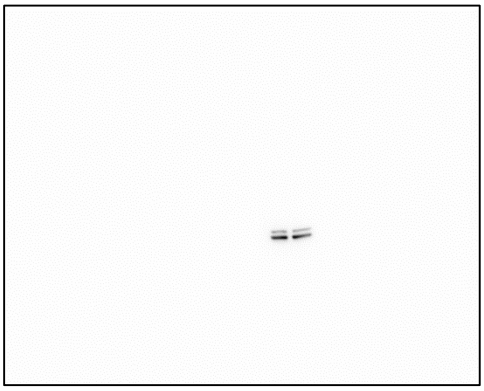

Erk1/2 (42,44KD)

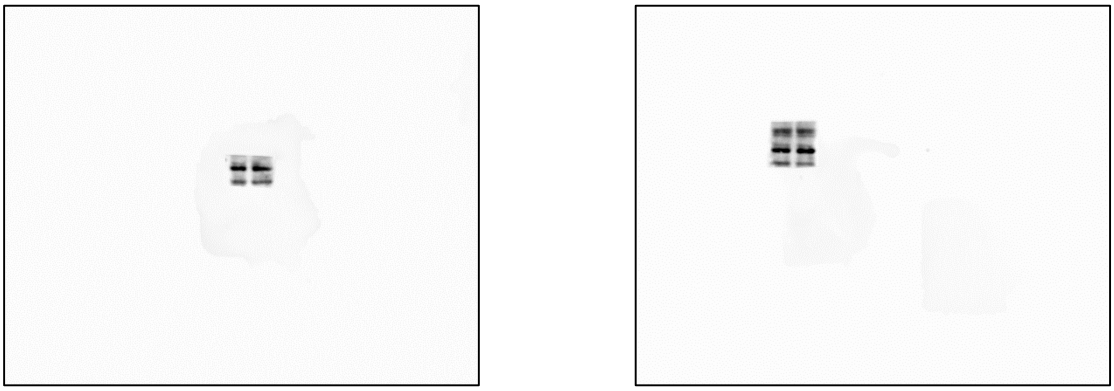

P-JNK (46,54KD)

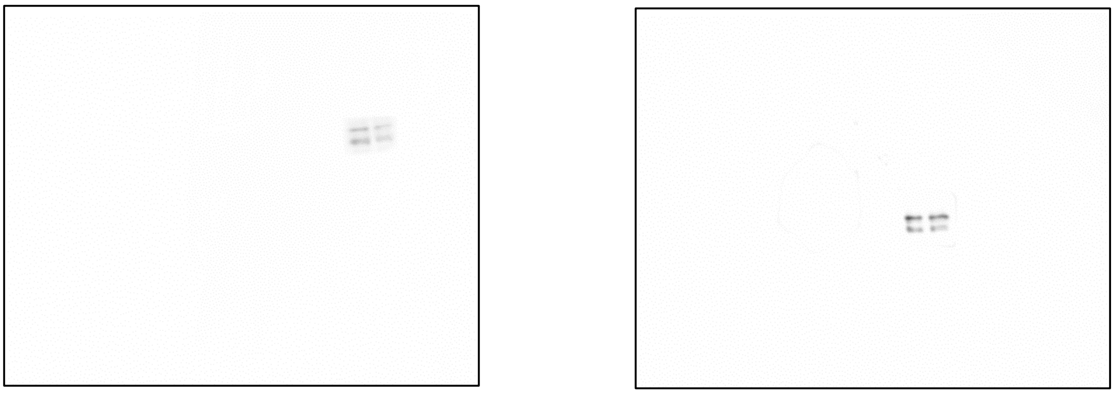

JNK (46,54KD)

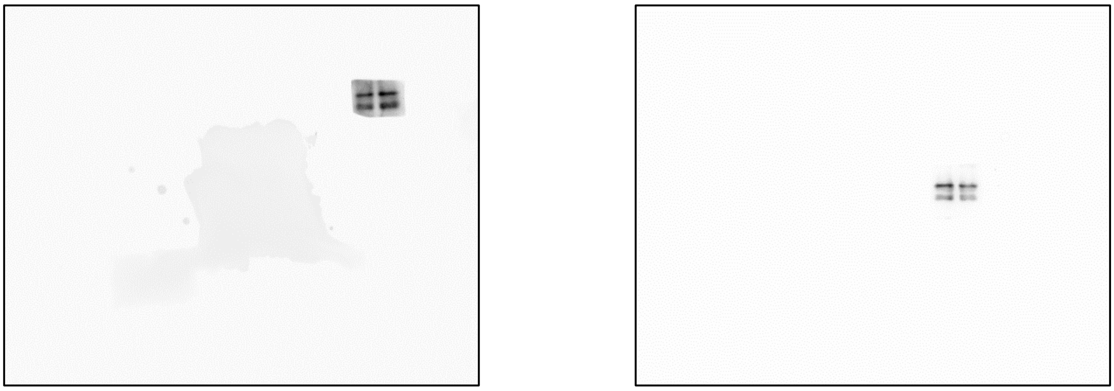

p-P38 (40KD)

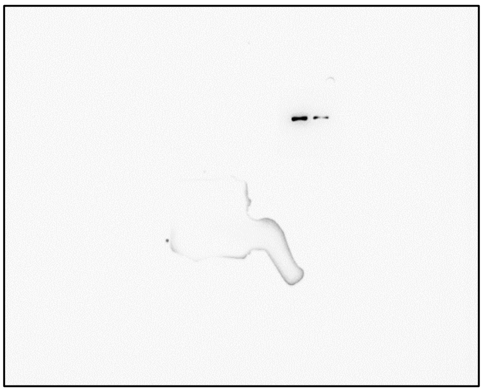

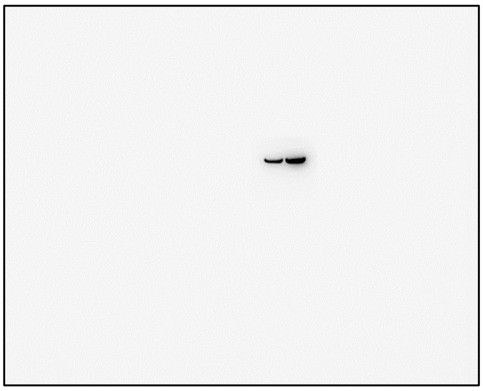

P38 (40KD)

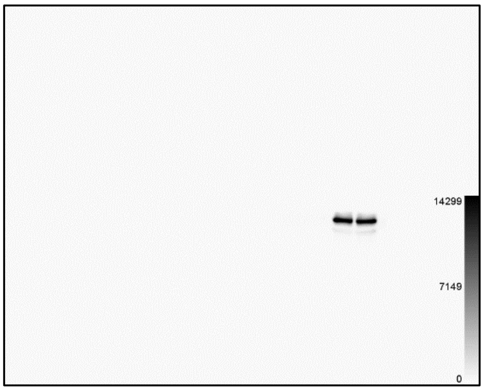

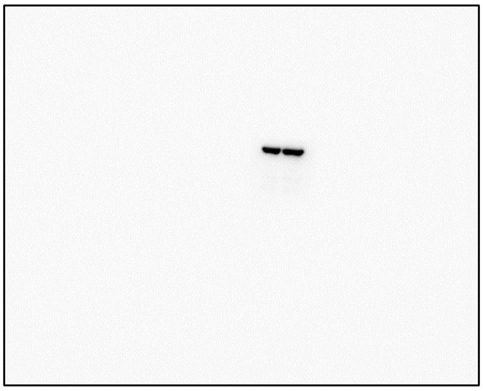

TDP2 (41KD)

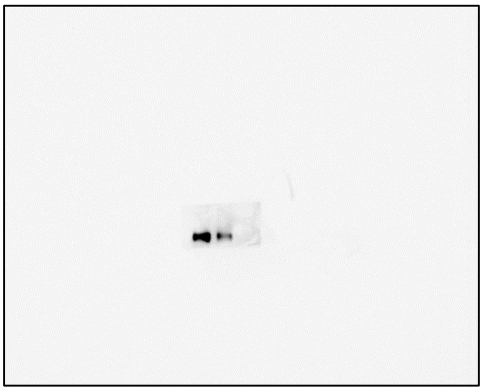

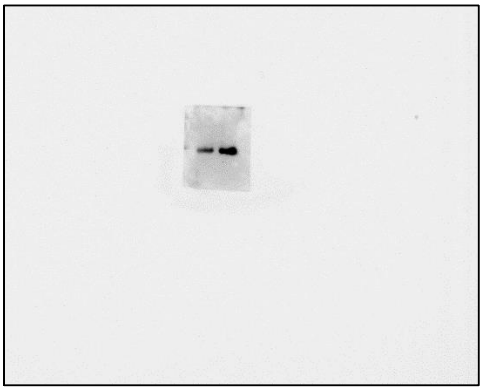

GAPDH (37KD)

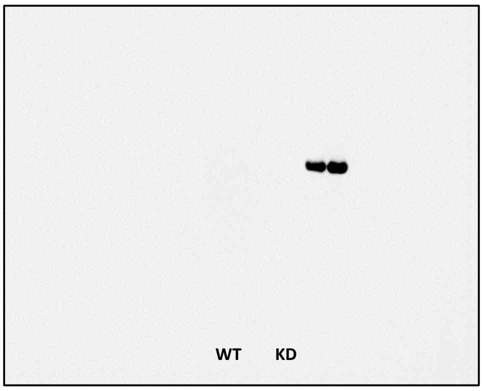

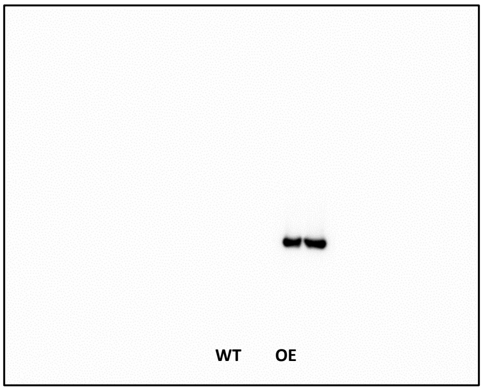

Supplementary Figure 1A

TDP2 (41KD)

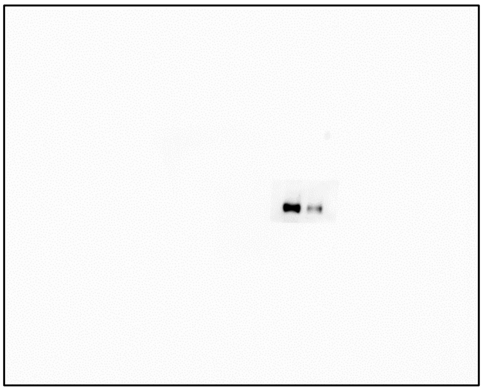

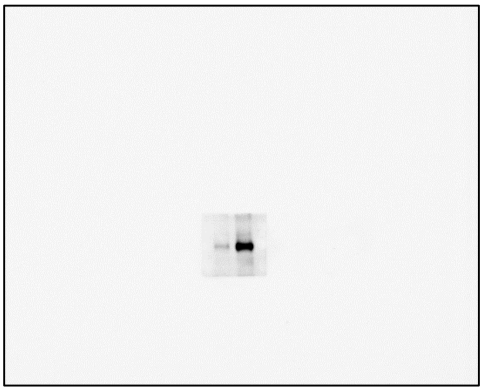

GAPDH (37KD)

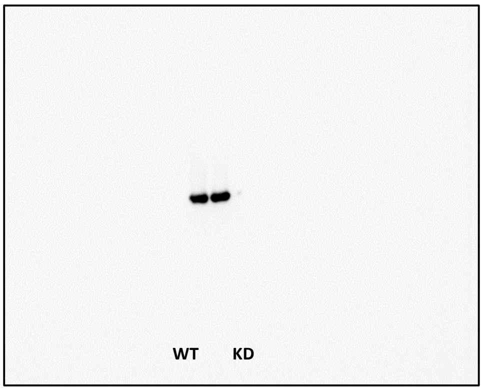

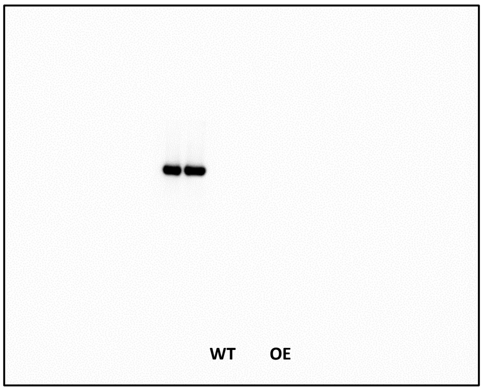

Supplementary Figure 1B

TDP2 (41KD)

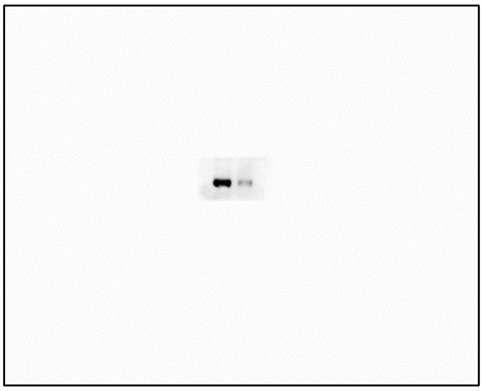

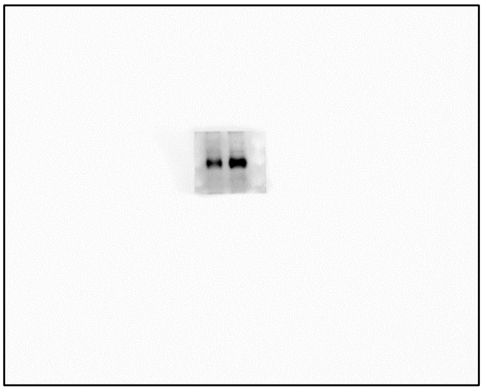

GAPDH (37KD)

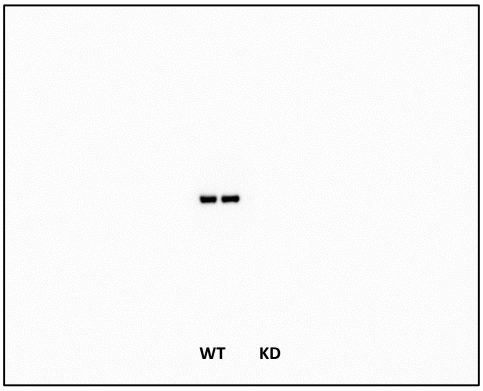

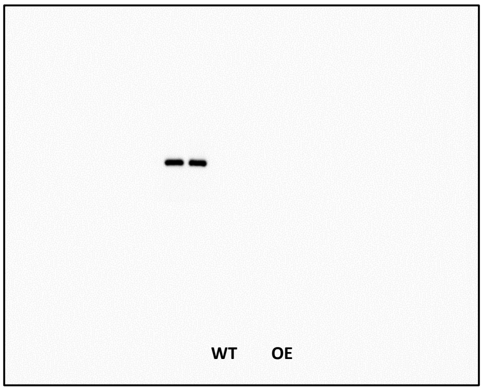

Supplementary Figure 3A

p-P38 (40KD)

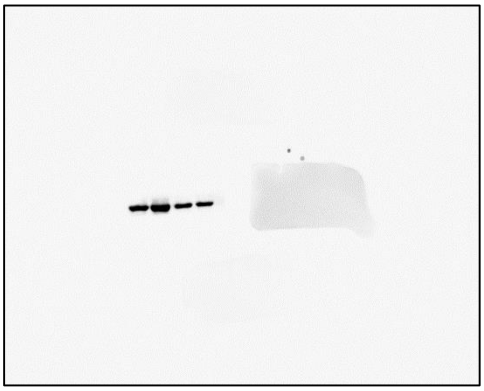

P38 (40KD)

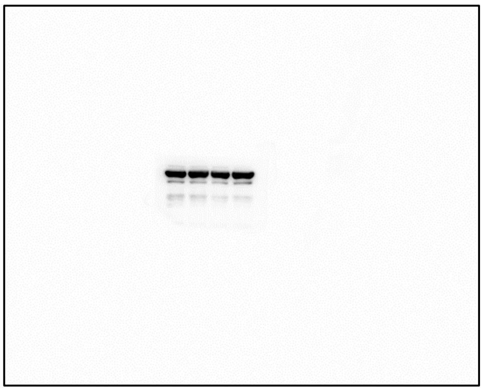

GAPDH (37KD)

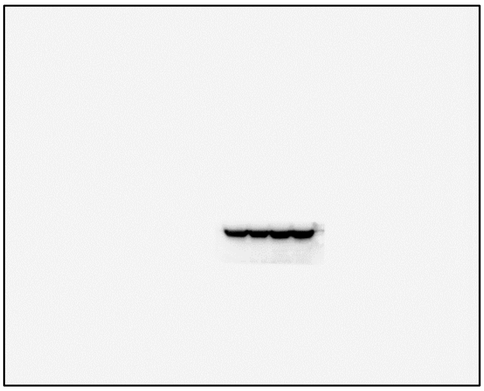

Supplementary Figure 3B

p-P38 (40KD)

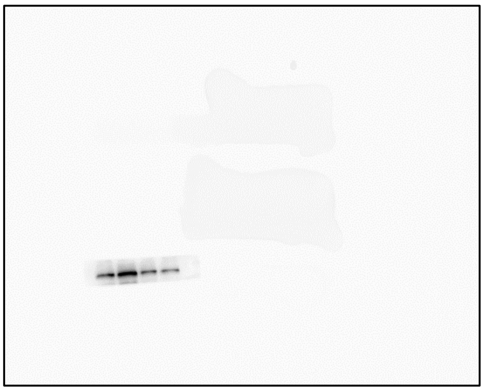

P38 (40KD)

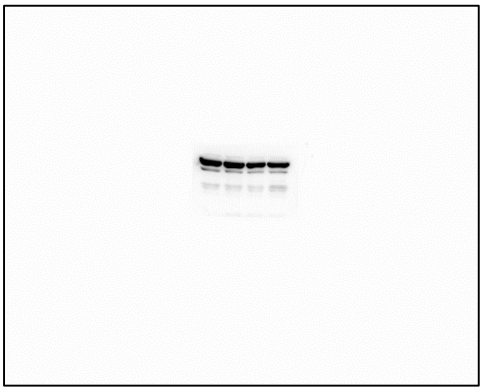

GAPDH (37KD)

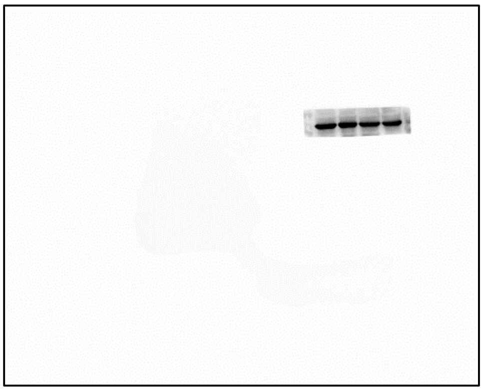

Supplement: S1 Raw Image — (PDF) [file pone.0339607.s012.pdf]
